# BZR1 Regulates Brassinosteroid-Mediated Activation of *AMT1;2* in Rice

**DOI:** 10.3389/fpls.2021.665883

**Published:** 2021-06-17

**Authors:** Shuo Yang, Depeng Yuan, Yang Zhang, Qian Sun, Yuan Hu Xuan

**Affiliations:** College of Plant Protection, Shenyang Agricultural University, Shenyang, China

**Keywords:** brassinosteroids, ammonium uptake, BZR1, *AMT1;2*, rice

## Abstract

Although it is known that brassinosteroids (BRs) play pleiotropic roles in plant growth and development, their roles in plant nutrient uptake remain unknown. Here, we hypothesized that BRs directly regulate ammonium uptake by activating the expression of rice *AMT1*-type genes. Exogenous BR treatment upregulated both *AMT1;1* and *AMT1;2* expression, while this induction was impaired in the BR-receptor gene *BRI1* mutant *d61-1*. We then focused on brassinazole-resistant 1 (*BZR1*), a central hub of the BR signaling pathway, demonstrating the important role of this signaling pathway in regulating *AMT1* expression and rice roots NH_4_^+^ uptake. The results showed that BR-induced expression of *AMT1;2* was suppressed in *BZR1 RNAi* plants but was increased in *bzr1-D*, a gain-of-function *BZR1* mutant. Further EMSA and ChIP analyses showed that BZR1 bound directly to the BRRE motif located in the promoter region of *AMT1;2*. Moreover, cellular ammonium contents, ^15^NH_4_^+^ uptake, and the regulatory effect of methyl-ammonium on root growth are strongly dependent on the levels of *BZR1*. Overexpression lines of *BRI1* and *BZR1* and Genetic combination of them mutants showed that BZR1 activates *AMT1;2* expression downstream of BRI1. In conclusion, the findings suggest that BRs regulation of NH4^+^ uptake in rice involves transcription regulation of ammonium transporters.

## Introduction

Inorganic nitrogen (N) is an important plant nutrient and is absorbed from the rhizosphere in two forms, nitrate and ammonium. In the paddy field, high levels of NH_4_^+^ are of particular importance to the rice yield. Further understanding of the molecular basis and regulation of ammonium transport and its translocation to buds is needed to promote efficient nitrogen absorption and to improve crop yields. Ammonium transporter (AMT) proteins can induce high-affinity NH4+ uptake from the rhizosphere to root cells, and the transporter AMT2;1 has also been shown to play a crucial role in ammonium root-to-stem translocation (Giehl et al., 2017). Sequence analysis has identified 10 AMT members in the rice genome (Suenaga et al., 2003; Loque and von Wiren, 2004). Of these, AMT1;1, AMT1;2, and AMT1;3 are the main three AMTs. The expression patterns of these three proteins differ between different tissues with *AMT1;2* and *AMT1;3* mainly expressed in plant roots, while *AMT1;1* is constitutively expressed in different tissues (Sonoda et al., 2003a). In rice, overexpression of *AMT1;1* increases NH_4_^+^ uptake, improves plant growth and promotes yield production in limited N-fertilization conditions (Ranathunge et al., 2014). However, the overexpression of *AMT1;3* has an opposite effect in regulating rice growth and NH_4_^+^ uptake to *AMT1;1* (Bao et al., 2015). Studies have shown that transcriptional regulation of *AMT* genes strongly influences the plant’s N content and uptake of different forms of externally applied N. NH_4_^+^ can upregulate the expression of both *AMT1;1* and *AMT1;2*, and inhibit the expression of *AMT1;3*. Under N starvation conditions, *AMT1;3* is upregulated in response to NH_4_^+^ (Kumar et al., 2003; Sonoda et al., 2003a). Similar regulation at the posttranscriptional and posttranslational levels have also been observed in *Arabidopsis*. For example, AMT1;1 has been shown to be phosphorylated in C-terminal threonine residue to inhibit transporter activity in an NH4^+^-dependent manner (Yuan et al., 2007; Lanquar et al., 2009), and further, CBL-interacting serine/threonine protein kinase 23 (CIPK23) was reported to phosphorylate AMT1 to inhibit ammonium uptake (Straub et al., 2017). Indeterminate domain 10 (IDD10), a transcription factor, has recently been shown to directly activate *AMT1;2* in rice (Xuan et al., 2013), furthermore, the ABI3/VP1 transcription factor RAVL1 activates *AMT1;2* to directly modulate NH_4_^+^ uptake in rice (Xuan et al., 2016).

Transcriptome studies using gain-of-function mutants of the BES1 transcription factor and wild-type plants have identified the *Arabidopsis* genes regulated by brassinosteroids (BRs). These genes included *AtAMT1;1* which was found to be upregulated by BR signaling activation (Goda et al., 2004; Yu et al., 2011). BRs are important phytohormones that bind to the cell surface receptor Brassinosteroid Insensitive 1 (BRI1), initiating a signaling cascade in which BRI1 binds to BRI1-Associated Receptor Kinase 1 (BAK1) leading to the downstream inactivation of the kinase Brassinosteroid Insensitive 2 (BIN2). The protein phosphatase PP2A dephosphorylates two master transcription factors Brassinozole-Resistant 1 (BZR1) and BRI1-EMS-Supperssor 1 (BES1), and the non-phosphorylated BZR1 and BES1 translocate to the nucleus to regulate the expression of BR responsive genes (Li and Chory, 1997; Li et al., 2002; Nam and Li, 2002; Kim and Wang, 2010; Yang et al., 2011; Guo et al., 2013; Tong and Chu, 2018). In addition, the stunting and BR-insensitive phenotype of *bri1* BR receptor mutants can be rescued by the enhanced stability of BES1 and BZR1 in *bes1-D* and *bzr1-D* gain-of-function mutants (Wang et al., 2002; Yin et al., 2002). In rice, RAVL1, an upstream component of BR signaling, regulates BR homeostasis through binding to an E-box motif in the promoter regions of the BR receptor and biosynthesis genes (Je et al., 2010). BRs treatment enhances the expression of *AMT1;1* and *AMT1;2* in rice (Xuan et al., 2016); however, the detailed mechanism remains obscure.

These findings raise the questions of whether BRs play a role in nutrient uptake, and whether there is a direct regulatory link between BZR1 and *AMT*s in rice. In this study, we first analyzed the expression of *AMT1* genes affected by BRs in the key BR signal transcription factor *BZR1* and the BR receptor gene *BRI1* mutants. We then investigated the expression patterns of *AMT1* family members in the roots of the *BRI1* mutant *d61-1*, *bzr1-D* siblings, as well as the *BZR1* knockdown *BZR1 RNAi*. Genetic combinations between *BRI1* and *BZR1* were generated to examine activation of BRI1 and BZR1 in BR-mediated induction of *AMT1;2*. In addition, the cellular ammonium contents and ^15^N abundance were tested to investigate BZR1 function in ammonium uptake. Taken together, our results showed that BR-dependent ammonium uptake is partially controlled by BZR1.

## Materials and Methods

### Plant Materials and Growth Conditions

The coding sequence of *BZR1* was cloned into the pCambia1302 vector to construct the BZR1-GFP-expressing plasmid. The pCambia1302-*BZR1* vector was subsequently transformed into Nipponbare rice calli to generate *BZR1-GFP* transgenic lines. The *bri1-D* in the Dongjin background as well as *d61-1*, *bzr1-D*, and *BZR1 RNAi* in the Nipponbare background were described previously (Yamamuro et al., 2000; Jeong et al., 2002; Bai et al., 2007; Qiao et al., 2017). *BZR1 RNAi*, *d61-1*, *bzr1-D*, *bri1-D*, *bri1-D*/*BZR1 RNAi*, *BZR1-GFP*, and *d61-1*/*bzr1-D* plants were grown in the greenhouse. Plants were first grown in distilled water (dH_2_O) for 1 week and subsequently transferred to brassinolide (BL) solution for analyzing BR effects on *AMT1* expressions. Whole roots were harvested after 3 h of BL treatment. To examine the effects of NH_4_^+^ on the expression of *BZR1*, plants were grown in dH_2_O for 2 weeks before transfer to N-free nutrient solution for a further 3 days of growth (Abiko et al., 2005). The plants were then grown in the nutrient solution containing 0.5 mM (NH_4_)_2_SO_4_ at pH 5.5. The roots were sampled at 0 and 3 h after the transfer. To test the effects of methyl-ammonium (MeA) on root growth, we added 1 mM KNO_3_ as the only source of N and different concentrations of MeA to 0.5 × MS medium, and cultivated wild-type, *BZR1 RNAi*, and *bzr1-D* plants in the modified medium. The primary root length was measured and recorded on the sixth day.

### RNA Extraction and Quantitative RT-PCR Analysis

Cellular total RNA was isolated by using the TRIzol reagent (Takara, Dalian, LN, China), and the RNA was treated with RQ-RNase-free DNase (Promega, Madison, WI, United States) to eliminate genomic DNA contamination. The GoScript Reverse Transcription kit (Promega, Madison, WI, United States) was used to synthesize cDNA. Quantitative RT-PCR was performed using the Illumina Research Quantity software Illumina Eco 3.0 (Illumina, San Diego, CA, United States), and each gene expression was normalized against that of the *Ubiquitin* level. The primers used for qRT-PCR are listed in Table 1.

**TABLE 1:** Primer sequences used in this study.

**Primer**	**Sequence**
Ubiquitin F	CACGGTTCAACAACATCCAG
Ubiquitin R	TGAAGACCCTGACTGGGAAG
AMT1;1 F	AGTACGTCGAGGAGATCTAC
AMT1;1 R	ACGTCGTTCGTTCTGGATTG
AMT1;2 F	TAGACATGGCCTCCCATCTC
AMT1;2 R	TAAGCATGATGTTCATGGTG
AMT1;3 F	AGGAGTACGTCGAGCTGATC
AMT1;3 R	CTTGCTCCGGCGACTTTCTG
AMT1;2 P-F	GCTCGCGGGATGGCGATGCGCGCTC
AMT1;2 P-R	GACGCGCGTCAACACAGACTGTA
BZR1 RT-F	GGAGTTCGAGTTCGACAA
BZR1 RT-R	CTCGGCGTCGGCGCGAAATGA
BZR1 F	AAGCTTATGACGTCCGGGGCGGCGGCG
BZR1 R	GGATCCTTTCGCGCCGACGCCGAGCGTGAG
P1 F	AATTTCGCTGCCATTTCC
P1 R	AAGAAGGAAGCTAAAGGC
P2 F	GAGTTTAGTTCTTTTGAC
P2 R	AAACCTAGGAAATTGATG
P3 F	TTGGAAAAATAGACATAC
P3 R	CGTTTAGTGTTTGAATCG
P4 F	CATATTTGTTTGATTAAC
P4 R	TGTGATATAGGGGGCAAG
A F	GTCTCACCGGGCTGCGTGCGTACGCCGATA
A R	TATCGGCGTACGCACGCAGCCCGGTGAGAC
mA F	GTCTCACCGGGCTGTTTTTTTACGCCGATA
mA R	TATCGGCGTAAAAAAACAGCCCGGTGAGAC
B F	TGGTCGCATCGTCGTGCGTGAGCTGCCTATCG
B R	CGATAGGCAGCTCACGCACGACGATGCGACCA
mB F	TGGTCGCATCGTTTTTTTGAGCTGCCTATCG
mB R	CGATAGGCAGCTCAAAAAAACGATGCGACCA
D2-F	ATG TGA TAA CAG AGA CGC TGC GGT
D2-R	TGG TGA CCA AGT GGT GAA GGA AGA

### ChIP Assay

Rice calli (8 g) expressing *35S: BZR1:GFP*, and *35S:GFP* were used for the ChIP assay. A pre-immune serum was used for pre-absorption before immunoprecipitation, and an anti-GFP monoclonal antibody (Clontech, Takara Bio, Japan) was used for immunoprecipitation. The immunoprecipitated DNAs were analyzed by ChIP-PCR for identification of the BZR1 binding region. The immunoprecipitated DNA was normalized by each input DNA in ChIP-PCR (Je et al., 2010). The primers used for the ChIP-PCR are shown in Table 1.

### Electrophoretic Mobility Shift Assay (EMSA)

*BZR1* ORF sequences were sub-cloned into the pET28a (+) expression vector to produce His:BZR1 recombinant protein in *Escherichia coli* strain BL21 DE3 after 4 h of 0.5 mM IPTG treatment at 28°C. To perform EMSA, 1 μg of His:BZR1 protein and 40k cpm of the ^32^P-labeled DNA probes were used. The protocol was followed as previously described (Je et al., 2010). Primers used in the EMSA are listed in Table 1.

### Transcriptional Activity Analysis

The effector (*35S:BZR1*), reporters [*pAMT1;2* and BRRE (BR Responsive Element)-mutated promoter *mpAMT1;1*-GUS fusions] and an internal control (*35S:LUC*) were co-transformed into protoplasts from *Arabidopsis* for testing transcriptional activation (Yamaguchi et al., 2010). *35S:BZR1* was cloned into the *GAL4BD* region of the *p35S:GAL4BD* vector and 2.5 kb of normal and BRRE-mutated (in which CGTG^*T*^/_*C*_G was replaced by TTTTTT) *AMT1;2* promoters were cloned into the TATA region of the *p35S:TATA:GUS* vector (Xuan et al., 2013). PEG-mediated transformation and subsequent activity measurement were performed as previously described (Yoo et al., 2007).

### Determination of Intracellular Ammonium Contents

Cellular ammonium contents in rice roots were calculated by using an F-kit (Roche, Basel, Switzerland) following the manufacturer’s instructions (Oliveira et al., 2002).

### ^15^N Uptake Analysis

Wild-type, *BZR1 RNAi*, *d61-1*, *bzr1-D*, *bri1-D*, *bri1-D/BZR1 RNAi*, and *d61-1/bzr1-D* plants were cultivated for a 2-week nursery period in deionized water, following which the seedlings were transferred to N-free nutrient solution to continue culturing (Sonoda et al., 2003b). After culturing for 3 days, the protoplasmic absorption of ^15^NH_4_^+^ was analyzed. The detailed method for the calculation of ^15^NH_4_^+^ influx and the ratio of ^15^N to ^14^N in the total N pool was as previously described (Xuan et al., 2016).

### Statistical Analysis

Statistical analysis was performed with Prism 5 software (GraphPad, San Diego, CA, United States). All data were expressed as mean ± SE. Comparison between multiple groups was performed by using one-way ANOVA with values of *P* < 0.05 considered as significant, followed by Bonferroni’ s multiple comparison tests.

## Results

### BR Treatment Induces BRI1-Dependent AMT1 Transcription

**FIGURE 1:**
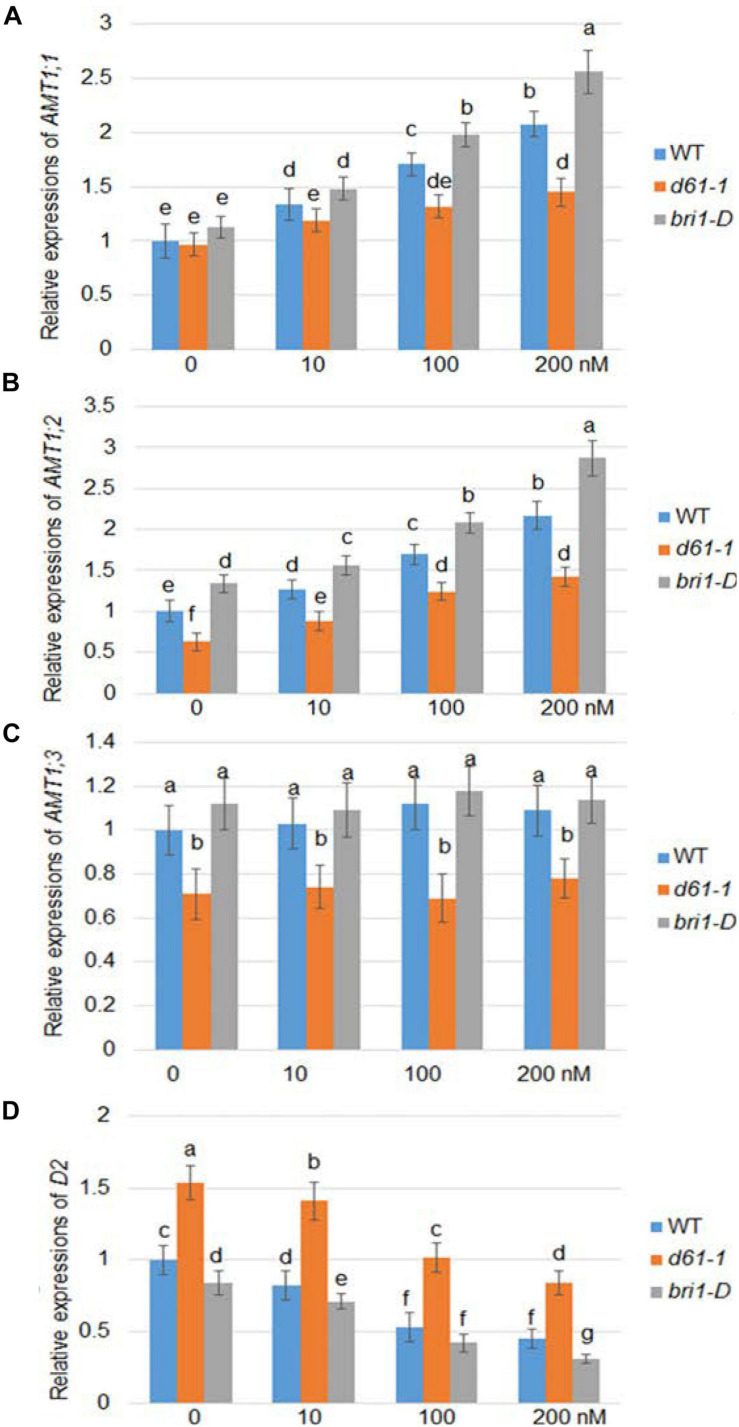
BR-dependent *AMT1* expression in wild-type, *BRI1* mutant *d61-1*, and *BRI1* overexpression line *bri1-D* plant roots. Rice plants were grown for 7 days in distilled water, followed by 3 h with 0, 10, 100, or 200 nM brassinolide (BL). The expression levels of *AMT1 1* **(A)**, *AMT1 2* **(B)**, *AMT1 3* **(C)**, and *D2* **(D)** in plants treated with BL was measured by qRT-PCR using *Ubiquitin* to normalize the expression of each gene. Error bars indicate means ± SE (*n* = 3). The experiments were repeated at least three times. Different letters represent statistically significant differences (*P* < 0.05).

Brassinolide is the most active form of BR. To investigate whether BR affects the expression of rice *AMT1*, we used a series of BL concentration gradients of 0, 10, 100, and 200 nm to treat the wild-type, a weak allele of rice *BRI1* mutant, *d61-1*, and the *BRI1* overexpression line *bri1-D* (Jeong et al., 2002), and compared the changes in the *AMT1* expression level under the different treatment conditions. Quantitative RT-PCR analysis showed different patterns in the response of the *AMT* gene to BR at the transcriptional level: *AMT1;1* and *AMT1;2* showed dose-dependent upregulation in response to BR treatment, while no obvious change was observed in *AMT1; 3*. Without BR treatment, the transcription levels of *AMT1;2* and *AMT1;3* in the *d61-1* mutant were slightly lower than those of the control group, while in *bri1-D*, the transcription levels of these genes were higher than those of wild-type plants. Among the various genotypes treated with BR, the expression levels of *AMT1;1* and *AMT1;2* in wild-type plants were higher than the level of *d61-1*. The expression level of *AMT1;1* and *AMT1;2* in *bri1-D* was significantly higher than that of the wild-type plants. Also, expression of a BR biosynthetic gene *D2* was analyzed. The result indicated that *D2* expression was suppressed by BR treatment in a dose-dependent manner, and *D2* expression level was higher in *d61-1* while lower in *bri1-D* compared to wild-type plants (Figure 1). These results suggest that BR mediates the regulation of *AMT1;1* and *AMT1;2* transcription levels via the cell surface receptor BRI1.

### BR-Mediated Induction of AMT1;2 Depends on BZR1

**FIGURE 2:**
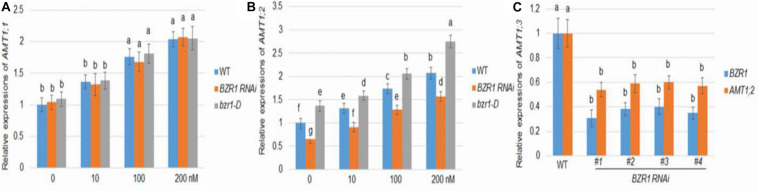
BR-mediated *AMT1* expression in *BZR1 RNAi* and BZR1 constitutively active *bzr1-D* plant roots. Rice plants were grown in distilled water for 7 days and then treated with 0, 10, 100, or 200 nM BL for 3 h. **(A)** The expression levels of *AMT1;1* was measured by qRT-PCR. **(B)** The expression levels of *AMT1;2* was measured by qRT-PCR. *Ubiquitin* was used to normalize the expression of each gene. Data represent means ± SE (*n* = 3). **(C)** *BZR1* and *AMT1;2* expression levels in wild-type and *BZR1 RNAi* lines (*#1*-*#4*). Data indicate means ± SE (*n* = 3). The experiments were repeated at least three times. Different letters represent statistically significant differences (*P* < 0.05).

As in *Arabidopsis*, rice BZR1 has been reported to be a key BR signaling transcription factor controlling the expression of downstream genes (Bai et al., 2007). To investigate the role of BZR1 in BR-mediated *AMT1* induction, *BZR1 RNAi* (*#1*), the knockdown transgenic line, and *bzr1-D* dominant mutant line were constructed (Bai et al., 2007; Ren et al., 2020). The changes in *AMT1;1* and *AMT1;2* transcription levels in these lines after BL treatment were monitored. The transcriptional abundance of *AMT1;1* was not affected by changes in BZR1 levels but could be increased in response to BL treatment in all plants (Figure 2A). Although the changes in the *AMT1;2* transcription level in the *BZR1 RNAi* mutants and *bzr1-D* were not significantly associated with BL treatment, they showed a significant decrease and increase, respectively. Only the *AMT1;2* expression levels correlated with the BZR1 level in a BR-dependent manner (Figure 2B). To verify *BZR1* levels in the *BZR1 RNAi* plants, qRT-PCR was performed. The results indicated that *BZR1* levels were reduced by about 60–70% in *BZR1 RNAi* lines (*#1*-*#4*). Also, *AMT1;2* expression levels were significantly lower in the *BZR1 RNAi* lines (*#1*-*#4*) than in the wild-type plants (Figure 2C).

### BZR1 Directly Binds to the Promoter to Activate AMT1;2 Expression

**FIGURE 3:**
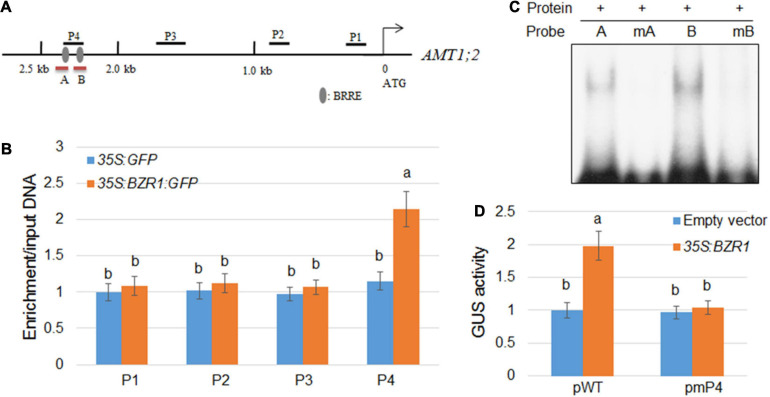
Activation of *AMT1;2* by BZR1 via binding to BRRE elements in its promoter region. **(A)** Schematic diagram showing the position of BRRE in the *AMT1;2* promoter and probes used for ChIP detection. The gray oval represents BRRE, the letters P (1, 2, 3, 4) indicate the positions of the probe. Red lines under the BRRE elements indicate the probe used in the EMSA analysis. **(B)** An anti-GFP antibody was used to amplify immunoprecipitated DNA in the 2.5 kb *AMT1;2* promoter (P1–P4) for CHIP detection. The qPCR method was used to determine the relative ratios of immunoprecipitated DNA to input DNA. Input DNA was used for data normalization. Data represent means ± SE (*n* = 3). Transgenic plants expressing GFP and BZR1-GFP under control of 35S promoter were used. Different letters represent statistically significant differences (*P* < 0.05). **(C)** The affinity of BZR1 to each BRRE elements (A and B) located in P4 region was evaluated by EMSA. The BRRE motif sequence (CGTG^*T*^/_*C*_G) was replaced with the sequence TTTTTT in the mA and mB probes, and the mutant BRRE probes (mA and mB) were used as the negative controls. **(D)** 35S:BZR1 and each GUS-expressing vector were subjected to transient co-expression experiments under the control of the natural and BRRE-mutated *AMT1;2* promoters. The activation of the native promoter (pWT) and BRRE element mutated promoter (pmP4) was measured in protoplasts that were co-transformed with *35S:BZR1*. The GUS expression data was normalized by the luciferase gene driven by the 35S promoter. Error bars are ± SE of the means (*n* = 3). The experiments were repeated at least three times. Different letters represent statistically significant differences (*P* < 0.05).

Since the changes in the transcription level of *AMT1;2* are highly consistent with the changes in the expression level of BZR1, which indicated that BZR1 acts as a transcriptional activator upstream of *AMT1;2*. To verify whether the binding site of BZR1 includes the promoter of *AMT1;2*, we constructed transgenic plants of *35S:GFP* and *35S:BZR1:GFP* and performed ChIP assays. Promoter-sequence analysis showed that two BRRE (BR Responsive Element) motifs were located within a 2.5 kb stretch in front of the *AMT1;2* start codon (Figure 3A). We designed four primer pairs to amplify the four fragments (P1−P4) of the *AMT1;2* promoter and performed qPCR experiments to check the GFP-immunoprecipitates in *35S:GFP* and *35S:BZR1:GFP* transgenic siblings (Figure 3B). The ChIP results showed that BZR1 could bind directly to the *AMT1;2* promoter, especially in the P4 region. Since the P4 fragment harbors two putative BRRE motifs (Figure 3A), we further performed EMSA experiments to determine which of the BRRE motifs was responsible for the BZR1 binding. We designed two specific probes each containing a BRRE motif. The B probe showed stronger binding to BZR1 (Figure 3A) while the binding of the A probe was slightly weaker. When the probes were mutated, their binding to BZR1 was lost. These results suggest that BZR1 can bind both BRRE motifs in the P4 region but the binding strength is different (Figure 3C).

In the current study, we identified two *cis*-elements targeted by the promoter of BZR1 which are closely related to transcriptional activation of *AMT1;2*. To further verify whether these *cis*-elements have similar functions *in vivo*, we used the *Arabidopsis* protoplast system to perform transient expression assays (Figure 3D). We also used the *35S:BZR1* plasmid and a vector expressing GUS, which is strictly controlled by one of four different types of 2.5 kb *AMT1;2* promoters namely, the native (pWT) and three mutated (pmP4) promoters. In *Arabidopsis* protoplasts, the *35S:BZR1* plasmid and the GUS vector are co-transformed into a vector expressing GUS. In these mutated promoters, BRRE motif sequences (CGTG^*T*^/_*C*_G) were observed to be replaced by the sequence TTTTTT. To eliminate the error caused by conversion efficiency, *35S:LUC* was used as an internal reference in each assay. By comparing the activity of the GUS genes under different promoter-driven conditions, we found that the GUS activity in *Arabidopsis* protoplasts was approximately twice that driven by the reporter gene promoter alone under the promoter pWT-driven conditions. Conversely, no detectable GUS activity was observed in the *Arabidopsis* protoplasts which were controlled by the mutant promoters (pmP4) (Figure 3D). These results indicated that direct combination with BZR1 is necessary for *AMT1;2* promoter activation.

### BZR1 Affects the Absorption of NH_4_^+^ by Plant Roots

**FIGURE 4:**
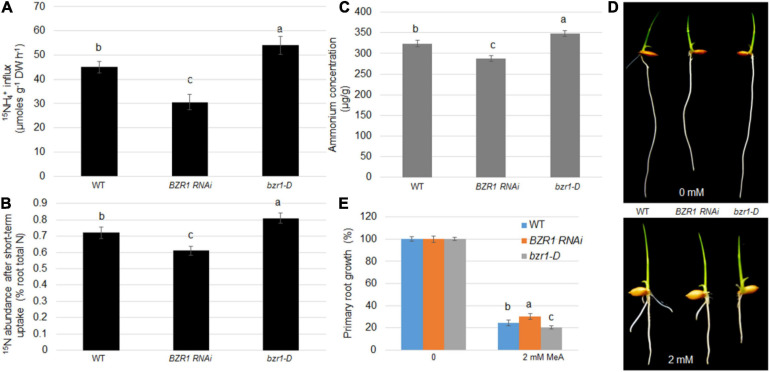
BZR1 effects on NH_4_^+^ uptake in plants. **(A)** The wild-type, *BZR1 RNAi* and BZR1 constitutively active *bzr1-D* plants were hydro-cultured in deionized water for 2 weeks, and then grown in a nitrogen-free nutrient solution for 3 days. After exposure to 200 μM ^15^N-labeled NH_4_^+^, the absorption of ammonium by rice roots was measured. Bars represent means ± SD (*n* = 6). **(B)** The wild-type, *BZR1 RNAi*, and *bzr1-D* plants were hydro-cultured in deionized water for 2 weeks, and then grown in a nitrogen-free nutrient solution for 3 days. After exposure to nutrient solution containing 200 μM ^15^N-labeled NH_4_^+^ for 6 min, the absorption of ammonium by rice roots and the ^15^NH_4_^+^ abundance in relation to the total ^15^N in roots was measured. Bars represent means ± SD (*n* = 6). **(C)** Intracellular NH_4_ levels of wild-type, *BZR1*, and *bzr1-D* grown for 3 days in 0.5 × MS were detected in plant roots. Data represent means ± SE (*n* = 3). **(D)** Under different conditions with 0 or 2 mM MeA, wild-type, *BZR1 RNAi*, and *bzr1-D* were grown in a modified 0.5 × MS medium containing 1 mM KNO_3_ for 6 days. MeA treatment significantly inhibited the growth of the primary root. In the absence of MeA treatment, the *BZR1 RNAi* primary root was shorter than that of the wild-type and *bzr1-D*. After 2 mM MeA treatment, bzr1-D showed a shorter primary root than *BZR1 RNAi* and wild-type. **(E)** The primary root growth was measured from wild-type, *BZR1 RNAi*, and *bzr1-D* with or without MeA supplementation as shown in **(D)**. Data represent means ± SE (*n* > 15 plants). The experiments were repeated at least three times. Different letters represent statistically significant differences (*P* < 0.05).

Root absorption of NH_4_^+^ is one of the main ways for plants to obtain N nutrients. To demonstrate whether BZR1 plays a role in this process, ^15^N-labeled ammonium was used to determine the efficiency of N absorption. The NH_4_^+^ concentrations in the root tissues of the wild-type, *BZR1 RNAi*, and *bzr1-D* lines were also determined. Seventeen day-old hydroponic seedlings were soaked in 200 μM of ^15^NH_4_^+^ solution for 6 min, after which the short-term import rate of the ^15^N-labeled ammonium in the plant roots was determined. Expressing the ^15^N influx in μmoles g^–1^ root dry weight h^–1^ (Yuan et al., 2007, 2013), the ^15^NH_4_^+^ influx in the *BZR1 RNAi* plants was only 68% of that of the wild-type, while the influx of ^15^NH_4_^+^ in the *bzr1-D* plants was greater than that of the wild-type plants (Figure 4A). We introduced the concept of “^15^N abundance,” representing the proportion of ^15^N to ^14^N in the total N pool, to further explore the role of BZR1 in the short-range transport of NH_4_^+^. Compared with the ^15^N internal flow results, the ^15^N abundance in the total ^15^N was lower in the *BZR1 RNAi* and higher in the *bzr1-D* plants compared to the wild-type (Figure 4B). The results of these short-term ^15^N absorption experiments indicate that BZR1 plays an important role in mediating NH_4_^+^ influx.

BZR1 can regulate the expression of *AMT1;2* genes in plant roots. We suspect that this regulation may be related to the long-term transport process of NH_4_^+^ in the roots. Therefore, the NH_4_^+^ content in the rice roots of 3-day-old wild-type, *BZR1 RNAi*, and *bzr1-D* seedlings grown on 0.5 × MS medium was determined. As expected, the content of NH_4_^+^ in the roots of *BZR1 RNAi* was lower than in the wild-type seedlings, while the roots of *bzr1-D* plants contained more NH_4_^+^ than the wild-type plants (Figure 4C). Furthermore, a toxic ammonium analog, MeA, was used as a replacement addition to the NH_4_^+^-free medium using a concentration gradient. Wild-type, *BZR1 RNAi*, and *bzr1-D* plants were grown in this medium and the length of the plant’s initial rooting was measured after 6 days of growth. In the absence of MeA, the primary roots of *BZR1 RNAi* were shorter than those of the wild-type and *bzr1-D*. With MeA treatment, the *BZR1 RNAi* root length was similar to that of the wild-type, while the *bzr1-D* roots were significantly shorter, the shortest in length among the three genetic lines (Figures 4D,E). These observations illustrated that the *BZR1 RNAi* response to MeA treatment is weaker than that of wild-type, while *bzr1-D* sensitivity to MeA treatment is relatively higher. Therefore, it can be inferred that BZR1 can participate in the process of rice root absorption of NH_4_^+^ in a long-term manner by regulating the expression of *AMT1;2* genes.

### BZR1 Regulates *AMT1;2* at the Downstream of BRI1

**FIGURE 5:**
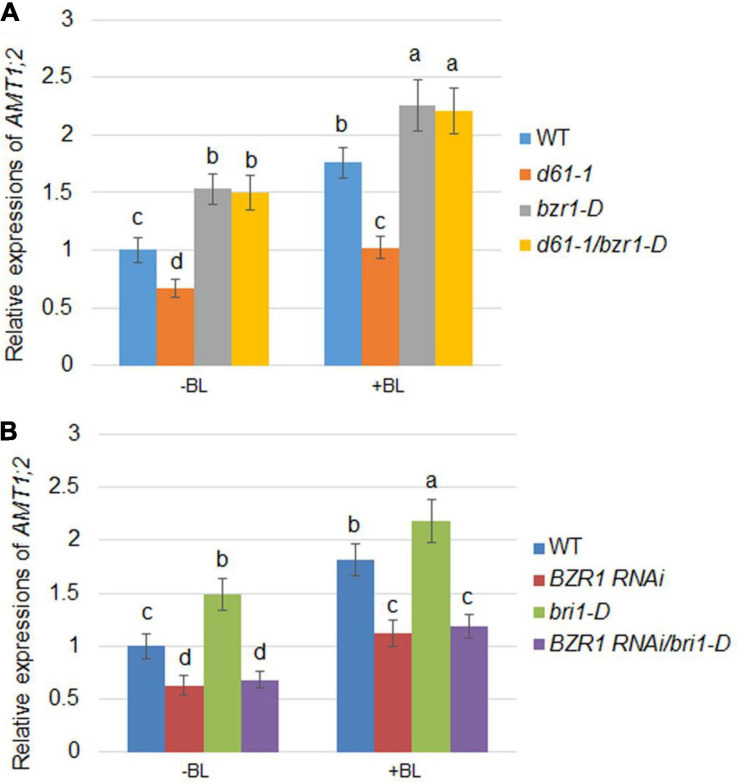
BR-induced *AMT1;2* expression in the genetic combinations between *BZR1* and *BRI1*. **(A)** The expression levels of *AMT1;2* genes in wild-type, *BRI1* mutant *d61-1*, gain-of-function mutant *bzr1-D*, and their cross offspring *d61-1*/*bzr1-D* before and 3 h after BL treatment. **(B)** The expression levels of *AMT1;2* genes in the wild-type, *BZR1 RNAi*, *BRI1* overexpression line *bri1-D*, and their cross offspring *BZR1 RNAi/bri1-D* before and 3 h after BL treatment. The above plants were grown in distilled water for 7 days with or without treatment of 100 nM BL for 3 h. The expression levels of *AMT1;2* genes were measured by qRT-PCR. Sample mRNA levels were normalized to those of *Ubiquitin*. Data represent means ± SE (*n* = 3). The experiments were repeated at least three times. Different letters represent statistically significant differences (*P* < 0.05).

As the expression of *AMT1;2* genes mediated by BR is inhibited in both the *d61-1* and *BZR1 RNAi* mutants, it is reasonable to speculate that *BZR1* and *BRI1* may affect the expression of *AMT1;2* genes. To investigate this, a series of genetic analyses were employed: *d61-1* and *bzr1-D*, *BZR1 RNAi*, and *bri1-D* lines were individually hybridized and two genetic combinations were constructed. We then measured the effects of BR-dependent *AMT1;2* genes expression in the *d61-1*, *bzr1-D*, *d61-1*/*bzr1-D*, and wild-type lines without or without BL treatment. High levels of *AMT1;2* mRNA were observed in both the *bzr1-D* and *d61-1*/*bzr1-D* lines after 3 h of root treatment with BL. In *d61-1*, the mRNA level was somewhat lower than in *d61-1*/*bzr1-D* but higher than the wild-type in both lines (Figure 5A). The mRNA levels of *AMT1;2* in three other genotypes, including *bri1-D*, *BZR1 RNAi*, and *bri1-D*/*BZR1 RNAi* plants, were subsequently determined. We observed a similar low-level expression of *AMT1;2* mRNA in *BZR1 RNAi* and a high-level expression in *bri1-D* plants with or without BR treatment. However, the *BZR1 RNAi* blocked *AMT1;2* induction in the *bri1-D* background (Figure 5B). These results demonstrated that BZR1 positively regulates *AMT1;2* expression downstream of *BRI1* irrespective of exogenous BR stimulation.

### Influence of BRI1 and BZR1 on NH_4_^+^-Dependent Expression of *AMT1;2* and NH_4_^+^ Uptake Activity

**FIGURE 6:**
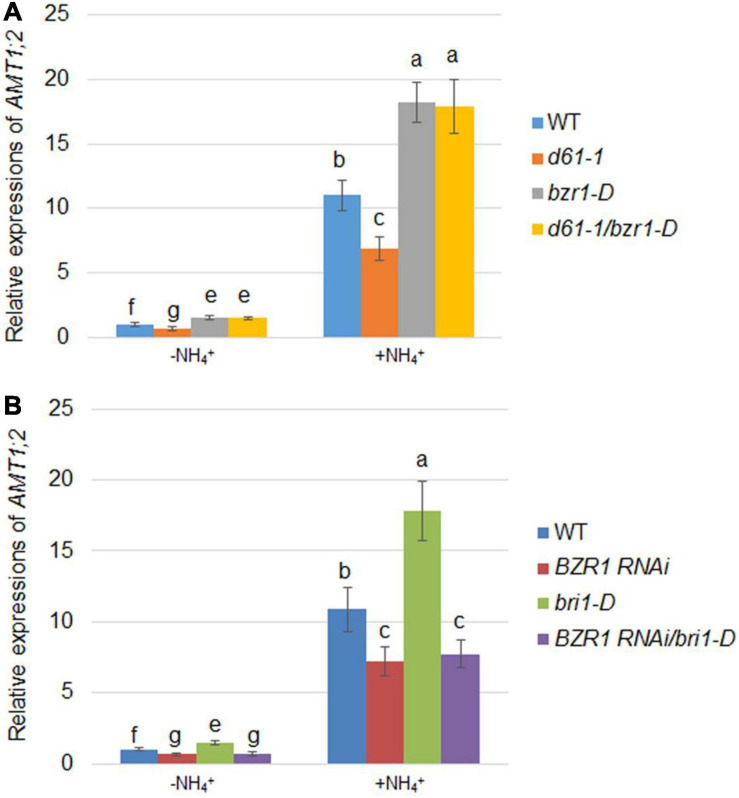
NH_4_^+^-induced *AMT1;2* expression in *BZR1*, *BRI1*, and their genetic combinations. **(A)** NH_4_^+^-induced *AMT1;2* expression was detected in the wild-type, *BRI1* mutant *d61-1*, gain-of-function mutant *bzr1-D*, and their cross offspring *d61-1*/*bzr1-D*. **(B)** NH_4_^+^-induced *AMT1;2* expression was detected in *BZR1 RNAi*, *BRI1* overexpression line *bri1-D*, and their cross offspring *BZR1 RNAi*/*bri1-D*. Full root sampling was carried out on 17-day-old seedlings that were transferred to nutrient solution containing 0.5 mM (NH_4_)_2_SO_4_ for 0 and 3 h. Measurement of *AMT1;2* expression levels using qRT-PCR. Sample mRNA levels were normalized with respect to those of *Ubiquitin*. Data represent means ± SE (*n* = 3). The experiments were repeated at least three times. Different letters represent statistically significant differences (*P* < 0.05).

Since *AMT1;2* is significantly regulated by NH_4_^+^, we assessed the effects of *BRI1* and *BZR1* on NH_4_^+^-induced *AMT1;2* expression. To determine the NH_4_^+^-dependent *AMT1;2* gene expression, we treated 17-day-old seedlings originally grown in dH_2_O and N-free nutrient medium with a 0.5 mM (NH_4_)_2_SO_4_ solution. The whole roots of the plant were then collected at 0 and 3 h after treatment. To examine whether *BZR1* and *BRI1* play the same role in NH_4_^+^-dependent expression of *AMT1;2* as in BR-dependent induction, *BZR1* and *BRI1* mutants were used to examine the *AMT1;2* expression levels. When compared with wild type, both *bzr1-D* and *d61-1*/*bzr1-D* contained higher mRNA levels of *AMT1;2*, while those in *d61-1* were lower (Figure 6A). Furthermore, the *bri1-D* lines showed high levels of *AMT1;2* mRNA, with reduced *AMT1;2* mRNA expression in *BZR1 RNAi*, regardless of NH_4_^+^ treatment. Nevertheless, similar patterns of mRNA expression of *AMT1;2* were observed in *BZR1 RNAi* and *bri1-D*/*BZR1 RNAi* (Figure 6B). Taken together, these results indicated that *AMT1;2* expression was sensitive to the BZR1 level and that BZR1 acts downstream of BRI1 in this signaling pathway.

**FIGURE 7:**
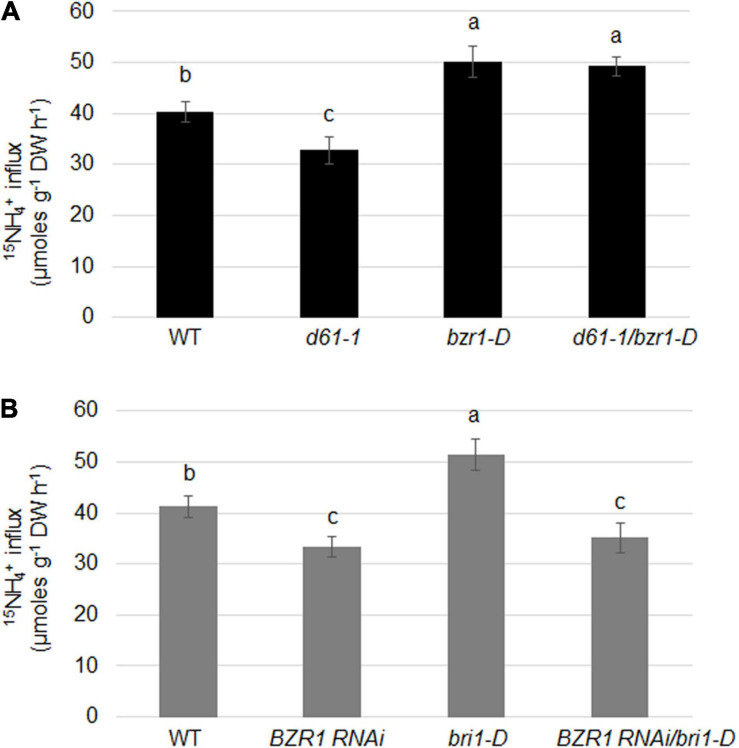
BRI1 and BZR1 effects on NH_4_^+^ uptake in plants. **(A)** ^15^NH_4_^+^ abundance in total ^15^N in roots of wild-type, *BRI1* mutant *d61-1*, gain-of-function mutant *bzr1-D*, and their cross offspring *d61-1*/*bzr1-D* plants. **(B)** ^15^NH_4_^+^ abundance in total ^15^N in roots of wild-type, *BZR1 RNAi*, *BRI1* overexpression line *bri1-D*, and their cross offspring *BZR1 RNAi*/*bri1-D* plants. The 2-week-old hydroponic seedlings were further transferred and cultured in nitrogen-free nutrient solution. Three days later, plant roots were immersed in 200 μM ^15^N-labeled NH_4_^+^ nutrient solution for 6 min. The rate of ammonium uptake by the roots was then determined. ^15^NH_4_^+^ abundance in total ^15^N in roots was measured after 6 min of ^15^NH_4_^+^ uptake. Bars represent means ± SD (*n* = 6). The experiments were repeated at least three times. Different letters represent statistically significant differences (*P* < 0.05).

To determine the effects of BRI1 and BZR1 on the absorption of NH_4_^+^ in plant roots, we examined the import of ^15^N-labeled ammonium in *BZR1* and *BRI1* genetic combinations. ^15^NH_4_^+^ influx was significantly lower in *d61-1*, while higher in *bzr1-D* or *d61-1*/*bzr1-D* than in the wild-type (Figure 7A). Also, the ^15^NH_4_^+^ influx was clearly lower in both *BZR1 RNAi* and *BZR1 RNAi/bri1-D*, while higher in *bri1-D* compared to wild-type plants (Figure 7B).

## Discussion

Although BRs are known for their pleiotropic roles in the regulation of plant growth and development, their effect on nutrient uptake is unclear. The activation of BR signaling has been shown by transcriptome analysis of BR treated wild-type or *bes1-D Arabidopsis* plants to positively regulate the expression of the AMT gene *AtAMT1;1* (Goda et al., 2004; Yu et al., 2011). Also, the BR signaling transcription factor RAVL1 activates *AMT1;2* to enhance NH_4_^+^ uptake in rice (Xuan et al., 2016). The possibility of a link between BRs and *AMT1* expression suggests that the process of root ammonium absorption may be coordinated with the physiological function of BRs in growth stimulation. In this study, we verified this issue by assessing the transcriptional levels of the AMT genes and the role they playing in ammonium uptake by the roots of rice lines, which genes involved in the regulation of BR homeostasis were modulated. We found high levels of BR susceptibility in all these genes which showed upregulated expression in response to BR treatment, especially the NH_4_^+^ transporter gene *AMT1;2*. Combining this result with the phenotype of higher NH_4_^+^ uptake, it can be inferred that this BR-dependent regulation is mediated by the direct binding between the transcription factor BZR1 and *AMT1;2* promoters. This regulation occurs after sensing BR signals. Thus, *AMT1;2*-mediated uptake of NH_4_^+^ can be seen as a physiological response mediated by BR.

BZR1 plays an important role in the coordination of ammonium uptake and BR signaling pathways. There are three lines of evidence that support this point. First, determination of the transcription level of *AMT1* genes in plants treated with exogenous BL showed that the *AMT1;2* in roots can respond to BL processing, increasing the level of transcription. BZR1 regulation plays a vital role in this process. Since the *AMT1;2* transcription level in *BZR1 RNAi* seedlings was also lower than that of the wild-type while the transcription level in the *bzr1-D* plants was higher; this expression pattern was also observed without BL treatment. Second, there was also a correlation between higher *AMT1;2* transcription levels and higher NH_4_^+^ uptake. We measured the rate of uptake of ^15^N-labeled NH_4_^+^ by rice roots expressing different BZR1 levels. NH_4_^+^ uptake rates were compared at ^15^N abundance and ^15^N influx levels, and it was found that the NH_4_^+^ uptake of *BZR1 RNAi* was significantly lower than that of the wild-type, which may be explained by down-regulation of *AMT1;2*. This concept is further supported by the findings in *bzr1-D* roots, where the enrichment of ^15^NH_4_^+^ was higher than that in the wild-type. Therefore, in the presence of ammonium, a non-significant increase in NH_4_^+^ influx may be related to the down regulation of *AMT* mRNA levels (Yuan et al., 2007) or AMT protein activity (Lanquar et al., 2009; Yuan et al., 2013) at posttranscriptional or posttranslational levels. Furthermore, increased *BZR1* expression levels were also associated with increased ammonium abundance in roots. We observed that all these NH_4_^+^ uptake-related traits were affected in these lines with or without additional BR treatment. This observation suggests that BZR1 is a positive regulator of NH_4_^+^ uptake under any condition conducive to plant growth. To sum up, BZR1 is the coordination center between NH_4_^+^ absorption and general growth promotion mediated by BRs. *AMT1;2* transcriptional level changes are significantly affected by BZR1 levels, suggesting a BZR1-dependent ammonium uptake pathway in roots. We have done further research on whether BZR1 directly regulates *AMT1;2* expression. Nine motifs were identified in the 2.5 kb promoter region of *AMT1;2* by promoter sequence analysis. EMSA, CHIP, and transient gene expression tests were used to further study the interaction between *AMT1;2* and BZR1. The two BRRE motifs in the promoter have been shown to be sites that bind BZR1 and directly activate *AMT1;2* transcription. In addition, although BZR1 can bind both BRRE motifs, the binding strength is different. It is likely that the positions of BRRE motifs in the promoter plays an important role in the regulation of *AMT1;2*. The comparison of BR- and NH_4_^+^-mediated expression of *AMT1;2* in *BZR1 RNAi*, *bzr1-D*, and wild-type plants indicates that constitutive activation of *AMT1;2* expression via BZR1 is independent of external signal stimulation. BRI1 performs a receptor function in BR signaling pathways and actively regulates BR-dependent *AMT1;2* expression through its signal reception. In addition, the roots of *d61-1*, the BRI1 mutant, accumulate less NH_4_^+^ than the corresponding wild-type, suggesting a positive role of BRI1 in regulating AMT-mediated NH_4_^+^ uptake in roots. The key BR signaling regulators BZR1, the BR receptor BRI1 play the putative role in the regulation of *AMT1;2* gene expression. Therefore, clarifying their inter-relationships enhances our understanding of the relationship between BR signaling and the mechanism of *AMT* regulation. We measured BR- or NH_4_^+^-mediated *AMT1;2* gene expression levels and ^15^NH_4_^+^ absorption in three rice lines with different combinations of *BZR1* and *BRI1* gene expression levels, including the line *bzr1-D* in the *d61-1* background and the line *bri1-D* in the *BZR1 RNAi* background. The activation of BRI1-induced *AMT1;2* requires BZR1 involvement, while the process of activation by BZR1 does not require BRI1 activity. Therefore, BZR1 plays a key role in coordinating root uptake of ammonium and BR-dependent plant growth gene expression regulation. Our study further elucidated a relationship between the two key factors BZR1 and BRI1 in the BR signaling pathway associated with regulation of *AMT1;2* gene expression and demonstrated that BZR1 was localized downstream of the *BRI1* during regulation of *AMT1;2* expression. It has been reported that BZR1 act as an integrator or master regulator to regulate plant growth, development, and immunity by directly interacting with key proteins from hormone signaling, stress signaling, cell elongation, flowering, immune signaling and so on (Eunkyoo et al., 2012; Lozano-Duran et al., 2013; Eunkyoo et al., 2014; Li and He, 2015; Nolan et al., 2017; Tian et al., 2018). However, whether BZR1 functions as heterodimer to regulate *AMT1;2* needs to be further analyzed.

Ammonium strongly induces expression of *AMT1;1* and *AMT1;2*, the two major AMTs in rice roots. However, the molecular mechanism of ammonium-mediated *AMT1* gene expression is not clear. The present results on the effect of BRI1 on NH_4_^+^-induction of these genes suggest a complex mechanism for the regulation of *AMT1* expression. Nonetheless, our results further support the existence of a common genetic component between plant nutrient uptake and the hormonal regulatory mechanisms involved in plant growth and development. In the current study, we found that although *AMT1;1* responded to BR treatment and its expression was induced in roots, BZR1 does not appear to have the ability to regulate *AMT1;1* expression. Different expression patterns were observed during the response of each member of the *AMT1* gene family to BR processing. These findings suggest that different BR-mediated regulatory circuits may have different effects on the same *AMT1* gene.

In addition, a recent study has shown that NH_4_^+^-induced miR444 positively regulates BR biosynthesis to regulate rice root growth. This microRNA targets five MADS-box transcription repressors directly upstream of *BR-deficient dwarf 1* (*OsBRD1*), a key BR biosynthetic gene (Jiao et al., 2020), suggesting NH_4_^+^ signaling also activates BR biosynthesis. Together with our results, we propose that there is a complicated feedback regulatory mechanism between NH_4_^+^ and BR signaling. It would be useful to investigate the effects of NH_4_^+^ on BZR1 protein levels or modifications in the future. Our study also found a highly complex interaction between plant hormone signaling and nutrient absorption pathways. These complex regulatory networks involved in ammonium uptake in plants require further genetic and molecular studies to elucidate their detailed mechanisms.

## Conclusion

Brassinosteroids play diverse functions in plant growth and development. In this study, we examined the role of BRs in ammonium uptake in rice. The data indicate that BR signaling activates *AMT1;1* and *AMT1;2* expression in the presence of the BR receptor BRI1, and the BR signaling transcription factor BZR1 directly activates *AMT1;2*. The further genetic study revealed that BZR1 activates *AMT1;2* expression downstream of *BRI1* to improve ammonium uptake in rice. These results indicate that BR signaling positively controls ammonium uptake partially via BZR1-mediated activation of *AMT1;2*, one of the key AMTs. Our analyses extend the knowledge of the BR-regulating module and its role in the regulation of nitrogen uptake in rice plants.

## Data Availability Statement

The original contributions presented in the study are included in the article/supplementary material, further inquiries can be directed to the corresponding author/s.

## Author Contributions

SY, YZ, and DY conceived this project. QS and YX provided the experimental design ideas and plant materials. YZ, SY, and DY carried out the experiments and generated the data. QS, YX, and DY contributed to the summary and analysis of the data. QS, YX, YZ, and SY wrote the manuscript. All authors contributed to the article and approved the submitted version.

## Conflict of Interest

The authors declare that the research was conducted in the absence of any commercial or financial relationships that could be construed as a potential conflict of interest.

## References

[B1] Abiko T. Obara M. Ushioda A. Hayakawa T. Hodges M. Yamaya T. (2005). Localization of NAD-isocitrate dehydrogenase and glutamate dehydrogenase in rice roots: candidates for providing carbon skeletons to NADH-glutamate synthase. *Plant Cell Physiol.* 46 1724–1734. 10.1093/pcp/pci188 16120687

[B2] Bai M. Y. Zhang L. Y. Gampala S. S. Zhu S. W. Song W. Y. Chong K. (2007). Functions of OsBZR1 and 14-3-3 proteins in brassinosteroid signaling in rice. *Proc. Natl. Acad. Sci. U.S.A.* 104 13839–13844. 10.1073/pnas.0706386104 17699623 PMC1959469

[B3] Bao A. Liang Z. Zhao Z. Cai H. (2015). Overexpressing of OsAMT1-3, a high affinity ammonium transporter gene, modifies rice growth and carbon-nitrogen metabolic status. *Int. J. Mol. Sci.* 16 9037–9063. 10.3390/ijms16059037 25915023 PMC4463577

[B4] Eunkyoo O. Zhu J. Y. Bai M. Y. Augusto A. R. Yu S. Wang Z. Y. (2014). Cell elongation is regulated through a central circuit of interacting transcription factors in the *Arabidopsis hypocotyl*. *eLife* 3:e03031. 10.7554/eLife.03031 24867218 PMC4075450

[B5] Eunkyoo O. Zhu J. Y. Wang Z. Y. (2012). Interaction between BZR1 and PIF4 integrates brassinosteroid and environmental responses. *Nat. Cell Biol.* 14 802–809. 10.1038/ncb2545 22820378 PMC3703456

[B6] Giehl R. F. H. Laginha A. M. Duan F. Rentsch D. Yuan L. von Wiren N. (2017). A critical role of AMT2;1 in root-to-shoot translocation of ammonium in *Arabidopsis*. *Mol. Plant* 10 1449–1460. 10.1016/j.molp.2017.10.001 29032248

[B7] Goda H. Sawa S. Asami T. Fujioka S. Shimada Y. Yoshida S. (2004). Comprehensive comparison of auxin-regulated and brassinosteroid-regulated genes in *Arabidopsis*. *Plant Physiol.* 134 1555–1573. 10.1104/pp.103.034736 15047898 PMC419831

[B8] Guo H. Li L. Aluru M. Aluru S. Yin Y. (2013). Mechanisms and networks for brassinosteroid regulated gene expression. *Curr. Opin. Plant Biol.* 16 545–553. 10.1016/j.pbi.2013.08.002 23993372

[B9] Je B. I. Piao H. L. Park S. J. Park S. H. Kim C. M. Xuan Y. H. (2010). RAV-Like1 maintains brassinosteroid homeostasis via the coordinated activation of BRI1 and biosynthetic genes in rice. *Plant Cell* 22 1777–1791. 10.1105/tpc.109.069575 20581303 PMC2910978

[B10] Jeong D. H. An S. Kang H. G. Moon S. Han J. J. Park S. (2002). T-DNA insertional mutagenesis for activation tagging in rice. *Plant Physiol.* 130 1636–1644. 10.1104/pp.014357 12481047 PMC166679

[B11] Jiao X. Wang H. Yan J. Kong X. Liu Y. Chu J. (2020). Promotion of BR biosynthesis by miR444 is required for ammonium-triggered inhibition of root growth. *Plant Physiol.* 182 1454–1466. 10.1104/pp.19.00190 31871071 PMC7054888

[B12] Kim T. W. Wang Z. Y. (2010). Brassinosteroid signal transduction from receptor kinases to transcription factors. *Annu. Rev. Plant Biol.* 61 681–704. 10.1146/annurev.arplant.043008.092057 20192752

[B13] Kumar A. Silim S. N. Okamoto M. Siddiqi M. Y. Glass A. D. (2003). Differential expression of three members of the AMT1 gene family encoding putative high-affinity NH4+ transporters in roots of *Oryza sativa* subspecies indica. *Plant Cell Environ.* 26 907–914. 10.1046/j.1365-3040.2003.01023.x 12803618

[B14] Lanquar V. Loque D. Hormann F. Yuan L. Bohner A. Engelsberger W. R. (2009). Feedback inhibition of ammonium uptake by a phospho-dependent allosteric mechanism in *Arabidopsis*. *Plant Cell* 21 3610–3622. 10.1105/tpc.109.068593 19948793 PMC2798313

[B15] Li J. Chory J. (1997). A putative leucine-rich repeat receptor kinase involved in brassinosteroid signal transduction. *Cell* 90 929–938. 10.1016/S0092-8674(00)80357-8 9298904

[B16] Li J. Wen J. Lease K. A. Doke J. T. Tax F. E. Walker J. C. (2002). BAK1, an *Arabidopsis* LRR receptor-like protein kinase, interacts with BRI1 and modulates brassinosteroid signaling. *Cell* 110 213–222. 10.1016/S0092-8674(02)00812-7 12150929

[B17] Li Q. F. He J. X. (2015). BZR1 interacts with HY5 to mediate brassinosteroid- and lightregulated cotyledon opening in *Arabidopsis* in darkness. *Mol. Plant* 9 113–125. 26363272 10.1016/j.molp.2015.08.014

[B18] Loque D. von Wiren N. (2004). Regulatory levels for the transport of ammonium in plant roots. *J. Exp. Bot.* 55 1293–1305. 10.1093/jxb/erh147 15133056

[B19] Lozano-Duran R. Macho A. P. Boutrot F. Segonzac C. Somssich I. E. Zipfel C. (2013). The transcriptional regulator BZR1 mediates trade-off between plant innate immunity and growth. *elife* 2:e00983. 10.7554/eLife.00983 24381244 PMC3875382

[B20] Nam K. H. Li J. (2002). BRI1/BAK1, a receptor kinase pair mediating brassinosteroid signaling. *Cell* 110 203–212. 10.1016/S0092-8674(02)00814-0 12150928

[B21] Nolan T. M. Brennan B. Yang M. Chen J. Zhang M. Li Z. (2017). Selective autophagy of BES1 mediated by DSK2 balances plant growth and survival. *Dev. Cell* 41 33–46. 10.1016/j.devcel.2017.03.013 28399398 PMC5720862

[B22] Oliveira I. C. Brears T. Knight T. J. Clark A. Coruzzi G. M. (2002). Overexpression of cytosolic glutamine synthetase. Relation to nitrogen, light, and photorespiration. *Plant Physiol.* 129 1170–1180. 10.1104/pp.020013 12114571 PMC166511

[B23] Qiao S. Sun S. Wang L. Wu Z. Li C. Li X. (2017). The RLA1/SMOS1 Transcription factor functions with OsBZR1 to regulate brassinosteroid signaling and rice architecture. *Plant Cell* 29 292–309. 10.1105/tpc.16.00611 28100707 PMC5354187

[B24] Ranathunge K. El-Kereamy A. Gidda S. Bi Y. M. Rothstein S. J. (2014). AMT1;1 transgenic rice plants with enhanced NH4(+) permeability show superior growth and higher yield under optimal and suboptimal NH4(+) conditions. *J. Exp. Bot.* 65 965–979. 10.1093/jxb/ert458 24420570 PMC3935567

[B25] Ren Y. Tian X. Li S. Mei E. He M. Tang J. (2020). *Oryza sativa* mediator subunit OsMED25 interacts with OsBZR1 to regulate brassinosteroid signaling and plant architecture in rice. *J. Integr. Plant Biol.* 62 793–811. 10.1111/jipb.12914 31990125

[B26] Sonoda Y. Ikeda A. Saiki S. von Wiren N. Yamaya T. Yamaguchi J. (2003a). Distinct expression and function of three ammonium transporter genes (OsAMT1;1-1;3) in rice. *Plant Cell Physiol.* 44 726–734. 10.1093/pcp/pcg083 12881500

[B27] Sonoda Y. Ikeda A. Saiki S. Yamaya T. Yamaguchi J. (2003b). Feedback regulation of the ammonium transporter gene family AMT1 by glutamine in rice. *Plant Cell Physiol.* 44 1396–1402. 10.1093/pcp/pcg169 14701935

[B28] Straub T. Ludewig U. Neuhauser B. (2017). The Kinase CIPK23 inhibits ammonium transport in *Arabidopsis thaliana*. *Plant Cell* 29 409–422. 10.1105/tpc.16.00806 28188265 PMC5354196

[B29] Suenaga A. Moriya K. Sonoda Y. Ikeda A. Von Wiren N. Hayakawa T. (2003). Constitutive expression of a novel-type ammonium transporter OsAMT2 in rice plants. *Plant Cell Physiol.* 44 206–211. 10.1093/pcp/pcg017 12610225

[B30] Tian Y. Min F. Qin Z. Lv H. Bai M. Y. (2018). Hydrogen peroxide positively regulates brassinosteroid signaling through oxidation of the BRASSINAZOLE-RESISTANT1 transcription factor. *Nat. Commun.* 9:1063. 10.1038/s41467-018-03463-x 29540799 PMC5852159

[B31] Tong H. Chu C. (2018). Functional specificities of brassinosteroid and potential utilization for crop improvement. *Trends Plant Sci.* 23 1016–1028. 10.1016/j.tplants.2018.08.007 30220494

[B32] Wang Z. Y. Nakano T. Gendron J. He J. Chen M. Vafeados D. (2002). Nuclear-localized BZR1 mediates brassinosteroid-induced growth and feedback suppression of brassinosteroid biosynthesis. *Dev. Cell* 2 505–513. 10.1016/S1534-5807(02)00153-3 11970900

[B33] Xuan Y. H. Duan F. Y. Je B. I. Kim C. M. Li T. Y. Liu J. M. (2016). Related to ABI3/VP1-Like 1(RAVL1) regulates brassinosteroid-mediated activation of AMT1;2 in rice (*Oryza sativa*). *J. Exp. Bot.* 68 727–737. 10.1093/jxb/erw442 28035023

[B34] Xuan Y. H. Priatama R. A. Huang J. Je B. I. Liu J. M. Park S. J. (2013). Indeterminate domain 10 regulates ammonium-mediated gene expression in rice roots. *New Phytol.* 197 791–804. 10.1111/nph.12075 23278238

[B35] Yamaguchi M. Ohtani M. Mitsuda N. Kubo M. Ohme-Takagi M. Fukuda H. (2010). VND-INTERACTING2, a NAC domain transcription factor, negatively regulates xylem vessel formation in *Arabidopsis*. *Plant Cell* 22 1249–1263. 10.1105/tpc.108.064048 20388856 PMC2879754

[B36] Yamamuro C. Ihara Y. Wu X. Noguchi T. Fujioka S. Takatsuto S. (2000). Loss of function of a rice brassinosteroid insensitive1 homolog prevents internode elongation and bending of the lamina joint. *Plant Cell* 12 1591–1605. 10.2307/3871176 11006334 PMC149072

[B37] Yang C. J. Zhang C. Lu Y. N. Jin J. Q. Wang X. L. (2011). The mechanisms of brassinosteroids’ action: from signal transduction to plant development. *Mol. Plant* 4 588–600. 10.1093/mp/ssr020 21471332

[B38] Yin Y. Wang Z. Y. Mora-Garcia S. Li J. Yoshida S. Asami T. (2002). BES1 accumulates in the nucleus in response to brassinosteroids to regulate gene expression and promote stem elongation. *Cell* 109 181–191. 10.1016/S0092-8674(02)00721-3 12007405

[B39] Yoo S.-D. Cho Y.-H. Sheen J. (2007). *Arabidopsis mesophyll* protoplasts: a versatile cell system for transient gene expression analysis. *Nat. Protoc.* 2 1565–1572. 10.1038/nprot.2007.199 17585298

[B40] Yu X. Li L. Zola J. Aluru M. Ye H. Foudree A. (2011). A brassinosteroid transcriptional network revealed by genome-wide identification of BESI target genes in *Arabidopsis thaliana*. *Plant J.* 65 634–646. 10.1111/j.1365-313X.2010.04449.x 21214652

[B41] Yuan L. Gu R. Xuan Y. Smith-Valle E. Loque D. Frommer W. B. (2013). Allosteric regulation of transport activity by heterotrimerization of *Arabidopsis ammonium* transporter complexes in vivo. *Plant Cell* 25 974–984. 10.1105/tpc.112.108027 23463773 PMC3634700

[B42] Yuan L. Loque D. Ye F. Frommer W. B. von Wiren N. (2007). Nitrogen-dependent posttranscriptional regulation of the ammonium transporter AtAMT1;1. *Plant Physiol.* 143 732–744. 10.1104/pp.106.093237 17172286 PMC1803739

